# Strategies to increase implementation of pharmacotherapy for alcohol use disorders: a structured review of care delivery and implementation interventions

**DOI:** 10.1186/s13722-019-0134-8

**Published:** 2019-02-12

**Authors:** Emily C. Williams, Theresa E. Matson, Alex H. S. Harris

**Affiliations:** 1 Health Services Research and Development (HSR&D) Center of Innovation for Veteran-Centered and Value-Driven Care, Veterans Affairs (VA) Puget Sound Health Care System, 1660 South Columbian Way, Seattle, WA 98108 USA; 2 Department of Health Services, University of Washington School of Public Health, Seattle, WA USA; 3 Kaiser Permanente Washington Health Research Institute, Seattle, WA USA; 4 Center for Innovation to Implementation, VA Palo Alto Health Care System, Menlo Park, CA USA; 5 Department of Surgery, Stanford University School of Medicine, Stanford, CA USA

**Keywords:** Alcohol use disorders, Medication assisted treatment, Pharmacotherapy, Naltrexone, Acamprosate, Disulfiram, Implementation strategies, Alcohol treatment

## Abstract

**Background:**

Effective medications for treating alcohol use disorders (AUD) are available but underutilized. Multiple barriers to their provision have been identified, and optimal strategies for addressing and overcoming barriers to use of medications for AUD treatment remain elusive. We conducted a structured review of published care delivery and implementation studies evaluating interventions that aimed to increase medication treatment for patients with AUD to identify interventions and component strategies that were most effective.

**Methods:**

We reviewed literature through May 2018 and used networking to identify intervention studies with AUD medication receipt reported as a primary or secondary outcome. Studies were identified as care delivery studies, characterized by patient-level recruitment and willingness to be randomized to candidate treatment options, and implementation studies, characterized by inclusion of all patients treated at sites involved in the study. Each identified study was independently coded by two investigators for strategies used, guided by a published taxonomy of implementation strategies. All authors reviewed coding discrepancies and revised codes based on consensus. After reaching internal consensus, we solicited feedback from lead investigators on studies to code additional strategies. We reviewed implementation strategies used across studies to assess their relationship with medication receipt, as well as alcohol use outcomes, as available.

**Results:**

Nine studies were identified: four RCTs of care delivery interventions, four quasi-experimental evaluations of large-scale implementation interventions, and one quasi-experimental evaluation of a targeted single-site implementation intervention. Implementation strategies used were variable across studies; no strategy was universally used. Effects of the interventions on receipt of AUD pharmacotherapy and alcohol use outcomes also varied. Three of four care delivery interventions resulted in increased receipt of AUD medications, but only one of these three improved alcohol use outcomes. One large-scale and one single-site implementation intervention were associated with increased AUD medication receipt, and these studies did not assess alcohol use outcomes. Patterns of implementation strategies did not clearly distinguish studies that successfully increased use of pharmacotherapy versus those that did not.

**Conclusions:**

Our review did not reveal strategies most effective for implementing AUD medications. Interventions designed to overcome identified barriers may have missed the mark, or differences in the intensity or targets of strategies may matter more than differences in strategies. Further research is needed to understand effective implementation methods and to better understand patient-level perspective, preferences and barriers to receipt of medications.

## Introduction

Alcohol use disorders (AUD) are common and associated with significant morbidity and mortality [1–3], but are substantially undertreated. In 2013, 16.6 million U.S. adults met diagnostic criteria for an AUD, but research suggests only 7.8% received any formal treatment [4]. One of the major gaps in treatment for AUD is the significant under-utilization of medications that are effective for treating AUD [1, 5, 6]. Three medications—*disulfiram*, *acamprosate*, and *naltrexone* (both oral and injectable)—have FDA approval specifically for the treatment of AUD, and *topiramate* has strong meta-analytic support [7]. Efforts to increase treatment of AUD with medications is motivated in part because the modality may address many reported barriers to receiving any formal AUD treatment [4, 8]. For instance, psychosocial treatments are often offered in group settings, heightening stigma-related issues for some patients, whereas medications can be provided on an individual basis [9]. In addition, patients may not be ready to abstain [8, 10]. Further, though this may be shifting over time [11, 12], many treatment programs view abstinence as the ultimate goal [8], whereas abstinence is not required with all medications and reduced drinking can be a goal of medication treatment [9]. Finally, AUD medications can be offered across healthcare settings, including primary care, which has been highlighted as an optimal setting for expansion of care for AUD [8, 13, 14].

Despite the promise of medication treatment for addressing several known barriers to AUD treatment and national recommendations encouraging medications be made available to all patients with AUD [15, 16], rates of pharmacotherapy for AUD remain extremely low. Among patients with AUD, 4-12% are treated pharmacologically [1, 6, 17–21]. Among subsets of patients with AUD and co-occurring schizophrenic, bipolar, posttraumatic stress or major depressive disorder, receipt of medications for AUD ranged from 7 to 11%, whereas receipt of medications for the comorbid disorder ranged from 69 to 82% [19]. This gap in the quality of AUD treatment is well known, and the substantial barriers to provision of AUD medications in diverse contexts have been described [22–27]. However, the optimal strategies for addressing these barriers and increasing use of medications for AUD treatment remain elusive.

In recent years, two related lines of research have contributed to knowledge regarding strategies to increase use of medications to treat AUD: evaluations of care delivery interventions and evaluations of implementation interventions. Care delivery interventions typically focus on improving patient-level clinical outcomes (e.g., reduction in heavy drinking days or abstinence from alcohol use), but often secondarily assess patient- or clinician-level process outcomes focused on treatment receipt (e.g., engagement in pharmacotherapy for AUD). Implementation interventions are typically designed to improve patient- or clinician-level process outcomes, but sometimes secondarily include patient-level clinical outcomes when the evidence for the effects of the underlying practice is weak (so called Hybrid I studies) [28]. Other key differences exist between these types of research that may influence both clinical and process outcomes. Most importantly, care delivery interventions typically involve recruitment of patients who are willing to be randomized to the treatment arms contained within the new care delivery model. Thus, these trials may be restricted to patients who are at least open to, if not actively interested in, treatment for AUD. On the other hand, evaluations of implementation interventions typically recruit and intervene on clinical entities (e.g., providers, clinics, hospitals) who serve large groups of patients who likely have more variable interest in treatment. Further, evaluations of care delivery interventions are typically designed to establish the effectiveness (or lack thereof) of particular care delivery models. Thus, these studies generally put significant effort and resources into ensuring fidelity to the care delivery model. On the other hand, implementation evaluations are often trying to establish the effectiveness of bundles of strategies (interventions) to increase uptake of practices that do not depend on external research resources. Thus, evaluations of implementation interventions may measure fidelity as a process outcome but typically exert less direct control [29].

Even though care delivery and implementation interventions differ in terms of methodology, patient inclusion criteria, and primary outcomes, they may evaluate the effectiveness of the same underlying implementation strategies, such as reorganizing, supplementing, or intervening on existing models of care [29]. The fact that the same component implementation strategies (e.g., audit and feedback) have been evaluated by these different research designs with very different patient populations affords an opportunity to take stock of the effectiveness of these interventions, and to distill insights into which designs, contexts, and component strategies appear to drive outcomes. Therefore, our goal was to conduct a structured review of published evaluations of care delivery and implementation interventions that have either primarily or secondarily aimed to increase use of pharmacotherapy for patients with AUD, with the goal of identifying component strategies that may be effective in increasing pharmacologic treatment of AUD. Our review was guided by an existing taxonomy of implementation strategies and terms identified via a three-round modified-Delphi process [30]. The purpose of our review was to learn which components have been tried most commonly and which strategies might be associated with larger effects. Also, due to the fact that evaluations of care delivery interventions exert greater efforts to ensure fidelity and include patients willing to be randomized, we hypothesized that higher adoption of medications for AUD will be observed in those contexts compared to implementation interventions, which typically aim to intervene on clinician and patient populations with greater variability in treatment motivation, knowledge, and preferences.

## Methods

For this structured literature review, we sought to identify published evaluations of care delivery and implementation interventions reporting effects on receipt of medication treatments for patients with AUD. We reviewed literature through May 2018. Studies were identified via searching PubMed, Google Scholar, and PsychInfo with relevant search terms (e.g., pharmacotherapy, alcohol use disorder medications, AUD medications, naltrexone, Acamprosate, disulfiram, medication-assisted treatment). We also reviewed reference lists from identified studies to identify additional studies that may have been missed by our search. Finally, because we have personally conducted and/or served as co-investigators on related studies, additional studies were also identified via networking. Once identified, each individual article was coded for implementation strategies used, as guided by Powell et al.’s refined compilation of implementation strategies resulting from the Expert Recommendations for Implementing Change (ERIC) project [30]. All articles were independently reviewed and coded by two investigators (EW and TM). When multiple articles and/or published protocols or commentaries were identified that described a single intervention and/or implementation effort, these articles were aggregated to the level of the intervention (e.g., three studies had adjoining published protocol papers, which were coded under the umbrella of a single study). Once coded, all authors met to review coding discrepancies, discuss interpretation of codes, arrive at consensus, and revise individual codes based on consensus.

After reaching internal consensus on coding, we reached out to the lead or senior author of each study to ask whether our codes aligned with their understanding/interpretation of their study and associated report. We shared Powell et al’s description of strategies and asked them to review our coding to see if they thought we had missed or miscoded anything. Finally, process (e.g., rates of prescribed AUD pharmacotherapy) and alcohol use outcome data were extracted from each study and described. All authors reviewed the coding of implementation strategies against study outcomes data to qualitatively identify sets of implementation strategies that might have been be most effective for increasing provision of AUD medications and report whether interventions that increased AUD pharmacotherapy also improved alcohol use outcomes.

## Results

Our literature review identified nine studies that evaluated interventions to primarily or secondarily increase utilization of pharmacotherapy for AUD. Four were randomized clinical trials of care delivery interventions designed to improve alcohol-related outcomes [31–38]. Four were quasi-experimental evaluations of large-scale implementation interventions designed to increase medication receipt [39–43], and one was a quasi-experimental evaluation of targeted implementation intervention in a single-site [44]. Two additional studies were identified but not included. The first reported on a large-scale implementation intervention designed to increase screening and brief intervention for unhealthy alcohol use and secondarily assessed whether the implementation was associated with increased receipt of AUD medications among those who screened positive [45]. However, it was not clear how many of the patients who screened positive met diagnostic criteria for AUD and thus would have been eligible for medication treatment, and, though findings regarding medication use were summarized, detailed data were not reported. The second report was a description of a demonstration project to implement extended release naltrexone in Los Angeles County, but no evaluation of the program’s effect on receipt of medication treatment among patients with AUD was reported [46].

Table 1 presents implementation strategies identified by our internal coding process across each identified study (labelled with X). All lead or senior authors of studies responded to our request for review of the codes and added additional codes (labelled with an O). Implementation strategies used were variable across the studies, and no strategy was used across all studies (Table 1). The most frequently used strategies were assessing readiness and identifying barriers and facilitators, distributing educational materials, facilitating relay of clinical data to providers (audit and feedback), and providing ongoing consultation. Strategies less frequently used involved payment and/or incentives or changes in laws and/or credentialing and licensing.

**Table 1:** Implementation strategies identified in published evaluations of care delivery and implementation interventions that have aimed to increase medication treatment for patients with alcohol use disorder

Strategy	Saitz AHEAD CCM [32]	Oslin Alcohol Care Management [31]	Watkins SUMMIT [35–38]	Bradley CHOICE [33, 34]	Robinson Group Manage [44]	Harris VA Academic Detailing Program [40]	Hagedorn ADaPT–PC [39, 42]	Ford Medication Research Partnership [43]	Ornstein PPRNet-TRIP [41]	Row total
Assess readiness and identify barriers/facilitators		O	X	X		X	X	X	X	7
Distribute educational materials		X	X	X		X	X	X	X	7
Facilitate relay of clinical data to providers	X	X	X	X		X	X		X	7
Provide ongoing consultation	O		X	X		X	X	X	O	7
Intervene with patients/consumers to enhance uptake and adherence	O	X	O		X		X	X		6
Conduct ongoing training		O	X			X	X	X	X	6
Create new clinical teams	X	X	X	X				X	O	6
Identify and prepare champions		O	X			X	X	X	X	6
Provide local technical assistance	O		O			X	X	X	X	6
Conduct educational meetings			X	X		X	X	X	X	6
Develop and implement tools for quality monitoring	X		X	X		X	X		X	6
Develop/organize quality monitoring systems	X	X	X			X	X			5
Conduct educational outreach visits			O			X	X	X	X	5
Audit and provide feedback	O	O				X	X		X	5
Develop educational materials		X	X			X	X		O	5
Organize clinician implementation team meetings	O		X				X	X	X	5
Facilitation			O			X	X	X	X	5
Obtain formal commitments			X			X	X	X	X	5
Remind clinicians	O		X			O	X		X	5
Revise professional roles	O		X		X			X	O	5
Provide clinical supervision	X	X	X	X						4
Develop academic partnerships			X			X		O	O	4
Promote adaptability	O		X				X		X	4
Centralize technical assistance		O				X	X		O	4
Conduct cyclical small tests of change			X			O		X	O	4
Create a learning collaborative			X				X	X	O	4
Make training dynamic		X		X				O	O	4
Purposely reexamine the implementation			O				O	X	X	4
Tailor strategies			X				X	X	X	4
Use an implementation advisor						X	O	O	O	4
Use data warehousing techniques		O				X	X		X	4
Conduct local needs assessment						X	X	X		3
Change record systems	O		O						X	3
Promote network weaving			X					X	O	3
Build a coalition							O	X	O	3
Conduct local consensus discussions			O					X	O	3
Develop a formal implementation blueprint			X			X			X	3
Recruit, designate, and train for leadership							X	O	X	3
Access new funding	O					X				2
Change service sites	X		X							2
Increase demand		X					X			2
Involve executive boards			O			O				2
Involve patients/consumers and family members							X	X		2
Prepare patients to be active participants	O						X			2
Use advisory boards and workgroups			O			O				2
Use data experts						X		?		2
Capture and share local knowledge								X	X	2
Identify early adopters						O			O	2
Make billing easier								X	O	2
Visit other sites						X			X	2
Alter incentive/allowance structures								X		1
Alter patient/consumer fees								X		1
Change physical structure and equipment	O									1
Inform local opinion leaders						X				1
Mandate change					X					1
Model and simulate change	X									1
Use train-the-trainer strategies							O			1
Fund and contract for the clinical innovation										0
Work with educational institutions										0
Develop resource sharing agreements										0
Change accreditation or membership requirements										0
Change liability laws										0
Create/change credentialing and/or licensure standards										0
Develop an implementation glossary										0
Develop disincentives										0
Obtain/use patient/consumer/family feedback										0
Place innovation on fee for service lists/formularies										0
Shadow other experts										0
Stage implementation scale up										0
Start a dissemination organization										0
Use capitated payments										0
Use mass media										0
Use other payment schemes										0

The effects of the interventions on receipt of AUD pharmacotherapy were also variable across studies (Table 2). In three of the four randomized evaluations of care delivery models [31–33], the interventions were associated with varying magnitude of increased receipt of AUD medications. At follow-up, treatment group rates of medication receipt ranged from 13 [36] to almost 70% [31]. The latter study, Oslin’s Alcohol Care Management model [31], was the only approach to significantly increase receipt of AUD medications and improve patient-level alcohol use outcomes (Table 2). Two of the four implementation interventions [40, 41] were associated with increased AUD medication receipt. While Ornstein’s Practice Partner Research Network-Translating Research Into Practice (PPRNet-TRIP) intervention appeared to have small early effects, proportions of patients receiving medications were so low that continued evaluation over time was not possible [41]. The Veterans Health Administration’s (VA) Academic Detailing Program appeared to increase rates of AUD medication receipt from 4.6 to 8.3% among patients with AUD [40]. Receipt of AUD medications also appeared to increase in in a single VA facility after implementation of a group medication management program attended by patients taking and considering medication treatment [44].

**Table 2:** Study designs and intervention effects on AUD medication receipt

Study (Author, abbreviated name, and reference)	Sample size (Patients/sites)	% Receiving AUD medications	Measure of AUD medication receipt	Intervention and intervention effect
SAITZ, AHEAD CCM [32]	563/1	*BASELINE*: Intervention 4% Control 8%	Receipt of addiction medication (buprenorphine, methadone, naltrexone, Acamprosate, disulfiram)	*Program Name and Brief Description*: The Addiction Health Evaluation and Disease (AHEAD) Management Chronic Care Management (CCM) model “included longitudinal care coordinated with a primary care clinician; motivational enhancement therapy; relapse prevention counseling; and on-site medical, addiction, and psychiatric treatment, social work assistance, and referrals (including mutual help). The control group received a primary care appointment and a list of treatment resources including a telephone number to arrange counseling.” AHEAD CCM was delivered by a multidisciplinary team (nurse care manager, social worker, internists, psychiatrist with addiction expertise) *Setting*: Hospital-based primary care practice (patients recruited from residential detoxification unit and referrals from urban teaching hospital) in Boston, MA *Goal*: Harm reduction *Key Components*: Use of registry to track and proactively reach out to patients, longitudinal care coordinated with primary care clinician and facilitated by shared electronic health record (EHR), motivational enhancement therapy, relapse prevention counseling, on-site medical, addiction and psychiatric treatment, social work assistance, and referrals (including to mutual help) *Effect on Medication Receipt*: OR = 1.88 (95% CI 1.28–2.75) *p* = 0.001 *Effect on Alcohol Use Outcomes*: Not significant
		*FOLLOW-UP*: Intervention 21% Control 15%		
OSLIN Alcohol Care Management [31]	163/3	*BASELINE*: Not reported	Receipt of naltrexone	*Program Name and Brief Description*: Alcohol Care Management “focused on the use of pharmacotherapy and psychosocial support. Alcohol Care Management was delivered in-person or by telephone within the primary care clinic. The control group received standard treatment in a specialty outpatient addiction treatment program” Delivered by a behavioral health provider in-person or over-the-phone with primary care provider recommendation and support. Behavioral health providers were trained in motivational interviewing *Setting*: Veteran Affairs (VA) primary care in New York and Philadelphia *Goal*: Abstinence *Key Components*: Weekly 30 min visits, individualized patient education, pharmacotherapy and psychosocial support, repeated assessment of alcohol use, encouraged treatment adherence, monitoring of problems and management of potential side effects, use of shared EHR for communication with primary care provider *Effect on Medication Receipt*: Naltrexone prescribed in 65.9% of the Alcohol Care Management group relative to 11.5% in control; Chi^2^ 50.10, *p* < 0.001 *Effect on Alcohol Use Outcomes*: The Alcohol Care Management group was more likely to refrain from heavy drinking than the control (OR = 2.16, 96% CI 1.27–3.66) but no effect on any alcohol use (OR = 1.40, 95% CI 0.75–2.59)
		*FOLLOW-UP*: Intervention 65.9% Control 11.5%		
WATKINS SUMMIT [35–38]	377/2	*BASELINE*: Not reported	Receipt of any “medication assisted treatment” with either long-acting injectable naltrexone or buprenorphine/naloxone.	*Program Name and Brief Description*: Collaborative care “was a system-level intervention, designed to increase the delivery of either a 6-session brief psychotherapy treatment and/or medication-assisted treatment with either sublingual buprenorphine/naloxone for opioid use disorders (OUDs) or long-acting injectable naltrexone for alcohol use disorders (AUDs). The control group was told that the clinic provided opioid and/or alcohol use disorder treatment and given a number for appointment scheduling and list of community referrals.” Delivered by care coordinators and therapists with a social work degree *Setting*: Primary care at Federally Qualified Health Center in L.A., CA *Goal*: Increase screening and brief intervention for unhealthy alcohol use *Key components*: Intended 6 sessions of brief psychotherapy and/or med-assisted treatment (buprenorphine/naloxone for OUD and naltrexone for AUDs), repeated assessments of substance use, use of registry to track and proactively reach out to patients, motivation and encouragement of engagement in therapy *Effect on Medication Receipt*: OR comparing intervention to control at 6-months follow-up for patients with AUD and/or OUD = 1.23 (95% CI 0.60–2.40) *p* = 0.53. Published commentary from SUMMIT investigators [37] suggests similar non-significant findings among patients with AUD only *Effect on Alcohol Use Outcomes*: Among patients with AUD only (54% of the sample) the SUMMIT intervention was significantly associated with abstinence from any alcohol use and all opioids at follow-up and was borderline significant for no heavy drinking in the past 30 days.
		*FOLLOW-UP*: Intervention 13.4% Control 12.6%		
BRADLEY CHOICE [33, 34]	304/3	*BASELINE*: Intervention 1% versus Control 2%	Receipt of naltrexone, Acamprosate or disulfiram	*Program Name and Brief Description*: Choosing Healthier Options in Primary Care (CHOICE) was a care management intervention in which “nurse care managers offered outreach and engagement, repeated brief counseling using motivational interviewing and shared decision making about treatment options, and nurse practitioner–prescribed AUD medications (if desired), supported by an interdisciplinary team (CHOICE intervention). The control group received usual primary care.” *Setting*: VA Primary care in Washington State *Goal*: Harm reduction *Key components*: Proactive outreach and engagement, repeated brief counseling using MI and shared decision-making about treatment options (AUD medication, biomarker assessment if abnormal baseline, behavioral goal-setting and skills development for reducing drinking, encouragement of mutual help and/or specialty addictions treatment, self-monitoring) *Effect on Medication Receipt*: OR = 6.3 (95% CI 3.4–11.8) *p* < 0.0001 *Effect on Alcohol Use Outcomes*: Not significant
		*FOLLOW-UP*: Intervention 32% versus Control 8%		
ROBINSON, Group Management [44]	1600/1	*BASELINE*: Increasing 0.08%/month in pre-implementation period	Receipt of naltrexone or Acamprosate, or extended-release naltrexone	*Program Name and Brief Description*: Group Management of pharmacotherapy initially implemented to provide continued access during a staffing shortage, sought to provide psychosocial education on medication management for alcohol dependence. Delivered by an addiction psychiatrist in collaboration with either an Addiction Therapist or a Certified Nurse Specialist *Setting*: VA San Diego Health Care System *Goal*: Increase adoption of pharmacotherapy for AUD *Key components*: Group participants capped at 8, review of naltrexone and Acamprosate, discussion of side effects or benefits, discussion of barriers to sobriety in group format. Sessions lasted 1 h *Effect on Medication Receipt*: The rate of increase in the percent of patients treated pharmacologically for alcohol dependence increased 0.08% per month in the pre-implementation period to 0.21% per month after group visits were implemented *Effect on Alcohol Use Outcomes*: Not measured
		*FOLLOW-UP*: Increasing 0.21%/month in post-implementation period		
HARRIS, VA Academic Detailing Program [40]	NA/37	*BASELINE*: Intervention 4.56% Control 6.01%	Monthly rates of receipt of naltrexone (oral or injectable), Acamprosate, disulfiram, or topiramate	*Program Name and Brief Description*: VA Academic Detailing Program in which “The academic detailers strove to educate, motivate, and enable key health care providers to identify and address the spectrum of hazardous alcohol use, especially to facilitate more active consideration of pharmacological treatment options for AUD.” Academic detailers were clinical pharmacy specialists *Setting*: VA medical centers and outpatient clinics in California, Nevada and the Pacific Islands *Goal*: Increase adoption of pharmacotherapy for AUD *Key components*: Local champions and leadership buy-in, dashboard for identifying patient candidates for AUD medication, repeated in-person visits to educate and build rapport with priority providers, problem-solve barriers and address knowledge gaps/misunderstanding about AUD meds, additional educational resources (e.g., patient education tools and pocket cards), integrated audit and feedback tools into EHR for identifying AUD patients, commitment from providers to increase prescribing patterns for AUD medication, monitoring and follow-up *Effect on Medication Receipt*: Slope of intervention sites increased more steeply than slope at control sites (*p* < 0.001). From immediately pre-intervention to the end of the observation period (Month 16–Month 36), the percent of patients with AUD who received medication increased 3.36% in absolute terms and 67.77% in relative terms *Effect on Alcohol Use Outcomes*: Not measured
		*FOLLOW-UP*: Intervention 8.32% Control 6.90%		
HAGEDORN, ADaPT–PC [39, 42]	NA/3	*BASELINE*: Intervention 3.8% at end of pre-implementation period Control 3.7%	Monthly rates of filled prescription for AUD medication (oral/injectable naltrexone, Acamprosate, disulfiram, topiramate) within 30 days after PC visit	*Program Name and Brief Description*: Alcohol Use Disorder Pharmacotherapy and Treatment in Primary Care settings (ADaPT–PC) “targets stakeholder groups with tailored strategies based on implementation theory and prior research identifying barriers to implementation of AUD pharmacotherapy. Local SUD providers and primary care mental health integration (PCMHI) providers are trained to serve as local implementation/clinical champions and receive external facilitation. Primary care providers receive access to consultation from local and national clinical champions, educational materials, and a dashboard of patients with AUD on their caseloads for case identification. Veterans with AUD diagnoses receive educational information in the mail just prior to a scheduled PC visit.” Delivered by site champions and external facilitators *Setting*: VA primary care *Goal*: Increase adoption of pharmacotherapy for AUD *Key components*: Training local champions, website with educational materials for primary care providers, a case-finding dashboard, technical assistance from local and national experts *Effect on Medication Receipt*: Rate of change (slope) increased significantly in the implementation period (*p* = 0.0023). Immediate post-implementation change not significant (*p* = 0.3401). Change over 12-month post-implementation relative to pre-implementation change significant (0.0033). No difference between intervention and control sites in immediate post-implementation change (p-0.8508). No difference between intervention and control sites in post-implementation slope (*p* = 0.4793) *Effect on Alcohol Use Outcomes*: Not measured
		*FOLLOW-UP*: Intervention 5.2% at end of implementation period Control 5.8%		
FORD Medication Research Partnership [43]	3887/9	*BASELINE*: Intervention 9.0% Control 11.4%	Receipt of AUD medication during an episode of care	*Program Name and Brief Description*: Medication Research Partnership, “a collaboration between a national commercial health plan and nine addiction treatment centers, implemented organizational and system changes to promote use of federally approved medications for treatment of alcohol and opioid use disorders.” Delivered by commercial health plan, “nationally recognized experts in the substance abuse field,” and “change leaders.” *Setting*: Specialty addiction treatment centers located on Northeastern seaboard of the U.S. *Goal*: Promote use of federally approved medications for AUD/OUD *Key components*: “Change leaders” and “change teams,” external coaches, rapid change cycles to test strategies to promote medication use, provider training and technical assistance *Effect on Medication Receipt*: Difference in differences at Year 3: Unadjusted: 5.8%; Adjusted: 5.2% (95% CI − 4.1 to 14.5) *p* = 0.27 *Effect on Alcohol Use Outcomes*: Not measured
		*3-YEAR FOLLOW-UP*: Intervention 26.5% Control 23.1%		
ORNSTEIN PPRNet-TRIP [41]	15053/19	*EARLY INTERVENTION CLINICS:* Phase 1: 2.6% Phase 2: 5.5%	Prescription for disulfiram, naltrexone (oral or injectable), Acamprosate, or topiramate	*Program Name and Brief Description*: Practice Partner Research Network-Translating Research Into Practice (PPRNet-TRIP) involved “practice site visits for academic detailing and participatory planning and network meetings for ‘best practice’ dissemination” *Setting*: Primary care practices from 15 U.S. States *Goal*: Increased prescription for disulfiram, naltrexone (oral or injectable), Acamprosate, or topiramate for those with an AUD *Key components*: EHR template, performance reports, provider education, and development of an implementation plan *Effect on Medication Receipt*: Due to small proportions of subjects receiving medications, pre-post (phase 1 versus phase 2) comparison of medication receipt was only estimable in the early intervention clinics. The adjusted OR for phase 1 versus phase 2 in the early intervention clinics was 2.24 (95% CI 1.03 -4.88) *p* < 0.05 *Effect on Alcohol Use Outcomes*: Not measured
		*DELAYED INTERVENTION CLINICS*: Phase 1: 0% Phase 2: 2.4%		

Patterns of implementation strategies did not clearly distinguish studies that successfully increased use of pharmacotherapy versus those that did not.

## Discussion

Nine studies have evaluated the effects of care delivery or implementation interventions designed to increase active consideration and use of pharmacologic treatment options for patients with AUD. The interventions varied widely in context, intensity, target populations, and the underlying strategies, though many strategies were shared across studies, regardless of design (care delivery or implementation intervention). As hypothesized, care delivery interventions, targeted on patients willing to be randomized, were associated with much larger and more consistent improvements in rates of medication receipt compared to implementation interventions targeted at the overall population of patients with AUD. Among the care delivery interventions evaluated, three out of four increased use of medications. However, of these three, only Oslin’s Alcohol Care Management intervention improved initiation of medications for AUD with more than one third of enrolled patients (69%) and improved in patient-level alcohol use. This trial may have been distinct from the others in its recruitment approaches—patients were recruited with the knowledge that the intervention aimed to provide pharmacologic treatment [31].

Among the implementation interventions evaluated, only the VA Academic Detailing Program [40] showed significant promise in increasing rates of medication receipt. It may be noteworthy that, compared to the other implementation interventions, the VA Academic Detailing Program was very labor intensive and targeted to diverse clinical settings with a high density of patients with AUD, not just primary care. The study of group medication management visits, intended as a means to increase prescribing capacity and educate patients who were considering medication treatment, [44] showed signals of effectiveness in one VA facility with a highly motivated champion. Interestingly, group settings have previously been identified as a barrier to receiving treatment for AUD, but appeared to facilitate increased treatment receipt among persons already seeking treatment. This intervention should be more rigorously evaluated in contexts where the primary barrier is low capacity to provide medication management.

A major goal of this review was to identify the underlying implementation strategies that were positively associated with larger effects. We categorized strategies based on published reports, but then solicited feedback from the intervention designers. There was substantial heterogeneity of strategies and some heterogeneity of effects, but no clear mapping of strategies to effectiveness was apparent. This process nonetheless proved informative by highlighting potential limitations of using of Powell et al.’s taxonomy to classify implementation strategies [30]. Specifically, strategies listed in the taxonomy appeared not be hermeneutically distinct, causing frequent difficulty classifying strategies as one or another. Relatedly, strategy definitions are somewhat inexplicit and hard to confidently map onto what was done in the interventions, resulting in different decisions being made by our two independent coders and between our coders and the lead or senior authors of publications. This discordance was greater when our team was not involved with the study and therefore had to rely on the published report to garner information. In all but one case, intervention developers added strategies to those identified by our 2-expert Delphi process. In some cases, the additional strategies were not fully described in the published reports. These findings suggest that an improved compilation of implementation strategies may be needed to enable accurate and reliable identification of distinct strategies. Efforts to refine such a compilation should consider designating umbrella strategies and sub-categories within them or providing a list of strategies that are similar but variable with regard to naming or minor procedural variants. Findings from our study also make clear the importance of comprehensive reporting of strategies used. While providing full descriptions of multi-faceted implementation strategies can be difficult in a single outcomes paper, authors should be encouraged to publish more detailed study protocols (as several did in the present study [34, 35, 38, 39]), and reviewers may, nonetheless, need to query intervention developers as a final validity check.

Perhaps more importantly, no method has been developed to characterize the intensity of strategies or cross-classify strategies with targets. Oslin’s Alcohol Care Management used many of the same strategies as other care delivery models but was targeted on patients willing to participate in an intervention focused on pharmacologic treatment. VA’s Academic Detailing Program did not differ from other implementation interventions in terms of component strategies so much as intensity and diversity of targets. Developing methods to more fully characterize interventions beyond component strategies may lead to insights that have greater utility for creating generalizable knowledge. In addition, because effectiveness of implementation interventions and strategies often depends on context, methods to cross-classify strategies with context and/or setting should be developed.

Beyond the aforementioned limitations of the existing implementation science tools used in this study, other limitations are worth noting. Although we searched multiple data sources and used reference lists from identified studies and networking to ensure comprehensive capture of existing studies, it is possible we missed intervention studies that aimed at increasing pharmacologic treatment of AUD. Second, our review identified only a small number of studies that reported receipt of AUD medication as a primary or secondary outcome. The small number of studies to date may limit the ability to identify generalizable information about the effectiveness of specific strategies. Moreover, of the nine studies that met inclusion criteria for this review, four were care delivery interventions tested in trials that were powered on main (clinical) outcomes. These studies may have been underpowered to detect differences in secondary outcomes, such as medication receipt.

Despite these limitations, this is the first review to our knowledge conducted with the goal of understanding strategies that may be effective for implementing medication treatment for AUD—a substantially underutilized treatment. Unfortunately, our review did not reveal which strategies are most effective for implementing AUD medications. However, we cataloged the use of specific strategies, perhaps suggesting candidates for future study. Further work is needed to understand why rates of medication treatment of AUD continue to be so low, even after patients are enrolled in care management interventions and/or receiving care in a healthcare setting that has been targeted by a multifaceted intervention. It is entirely possible that previous examinations of barriers, and interventions designed to overcome them have missed the mark. To further assess this, research will be needed to better understand patient-level perspective, preferences and barriers to receipt of medications.

## Abbreviations

VA: U.S. Veterans Health Administration
AUD: alcohol use disorder
ERIC: Expert Recommendations for Implementing Change project
PPRNet-TRIP: Practice Partner Research Network-Translating Research Into Practice
AHEAD: Addiction Health Evaluation and Disease
CCM: Chronic Care Model
EHR: electronic health record
SUMMIT: Substance Use Motivation and Medication Integrated Treatment
CHOICE: Choosing Healthier Drinking Options in Primary Care
ADaPT–PC: Alcohol Use Disorder Pharmacotherapy and Treatment in Primary Care Settings
OUD: opioid use disorder
PCMHI: primary care mental health integration
